# Pharmacist-Led Implementation of Brief Tobacco Cessation Interventions during Mobile Health Access Events

**DOI:** 10.3390/pharmacy11020072

**Published:** 2023-04-07

**Authors:** Karen Suchanek Hudmon, Julia S. Czarnik, Alexa M. Lahey, Susie J. Crowe, Megan Conklin, Robin L. Corelli, Jasmine D. Gonzalvo, Katy Ellis Hilts

**Affiliations:** 1 Department of Pharmacy Practice, College of Pharmacy, Purdue University, West Lafayette, IN 47907, USA; 2 Department of Clinical Pharmacy, School of Pharmacy, University of California San Francisco, San Francisco, CA 94143, USA; 3 Center for Health Equity and Innovation, Department of Pharmacy Practice, College of Pharmacy, Purdue University, Indianapolis, IN 46202, USA; 4 Richard M. Fairbanks School of Public Health, Indiana University, Indianapolis, IN 46202, USA

**Keywords:** pharmacy, pharmacist, tobacco, smoking, tobacco cessation, smoking cessation, vaccination

## Abstract

To address gaps in care for individuals from under-resourced communities disproportionately affected by tobacco use, this pharmacist-led demonstration project evaluated the feasibility of implementing tobacco use screening and brief cessation interventions during mobile health access events. A brief tobacco use survey was administered verbally during events at two food pantries and one homeless shelter in Indiana to assess the interest and potential demand for tobacco cessation assistance. Individuals currently using tobacco were advised to quit, assessed for their readiness to quit, and, if interested, offered a tobacco quitline card. Data were logged prospectively, analyzed using descriptive statistics, and group differences were assessed by site type (pantry versus shelter). Across 11 events (7 at food pantries and 4 at the homeless shelter), 639 individuals were assessed for tobacco use (n = 552 at food pantries; n = 87 at the homeless shelter). Among these, 189 self-reported current use (29.6%); 23.7% at food pantries, and 66.7% at the homeless shelter (*p* < 0.0001). About half indicated readiness to quit within 2 months; of these, 9 out of 10 accepted a tobacco quitline card. The results suggest that pharmacist-led health events at sites serving populations that are under-resourced afford unique opportunities to interface with and provide brief interventions for people who use tobacco.

## 1. Introduction

Tobacco use continues to be the leading known preventable cause of disease, disability, and death in the United States [1], with 12.5% of adults reporting current cigarette smoking and 19.0% reporting use of any type of tobacco product [2]. Although the overall prevalence of tobacco use has decreased over the past several decades, it varies widely across population subgroups [2], and it is well established that individuals from under-resourced groups, including those experiencing homelessness and/or food insecurity, are disproportionately affected by tobacco use and report poorer tobacco cessation outcomes [2,3,4,5]. Additionally, research has shown that individuals who smoke are at an elevated risk for poorer outcomes from influenza and COVID-19, including hospital admissions and progression to severe disease, compared to those who do not smoke [6,7]. While more than two thirds of people who smoke cigarettes express interest in quitting, for a variety of reasons, most do not use evidence-based approaches to help them quit [8]. The national surveys estimates that of adults who attempt to quit smoking, only 6.8% use behavioral counseling, 29.0% use medication, and 4.7% use both [8]. There are numerous barriers that under-resourced populations might face in accessing tobacco cessation services, such as transportation-related issues, access to a regular healthcare provider, and the out-of-pocket costs of care [9,10,11]. To address these barriers, increased access to tobacco cessation services for under-resourced groups is needed. One approach is to “layer” tobacco cessation on top of other existing services that are already being provided [5], thereby minimizing any additional burden for the provision of care (for the clinician) and time, travel, etc. (for the client).

Food banks and homeless shelters serve as key resources for populations that are under-resourced to receive immediate access to essential goods and services. Because food banks and shelters provide the public with assistance on most days of the week, they are viable alternative sites for the delivery of healthcare services. For example, there is evidence to suggest that screenings [12,13,14,15,16] and preventive care [15,16,17] delivered in such settings can be effective in identifying at-risk individuals and providing them with and/or connecting them to care. Of relevance to the current study, there is specific support for the delivery of tobacco cessation and other health services in food pantries and homeless shelters [15,18,19], as well as the role pharmacists can play as providers in these settings [16,19]. As such, pharmacist-led mobile health access events providing immunization services in food banks or homeless shelters can provide opportunities to reach individuals from under-resourced communities regarding tobacco cessation.

Building upon prior literature [20,21], the objective of this demonstration project was to evaluate the feasibility of providing brief tobacco cessation interventions that included a referral to the tobacco quitline during mobile health access events. Specifically, we assessed the potential demand for and interest in tobacco cessation services among participants as an indicator of feasibility [22]. These events were hosted as outreach efforts in Central Indiana in conjunction with pharmacists and pharmacy students through the Purdue University College of Pharmacy’s Center for Health Equity and Innovation (CHEqI) [23]. Findings from this project can be informative in guiding ongoing and future efforts related to pharmacists’ role in expanding access to preventive care and tobacco cessation services to under-resourced communities.

## 2. Materials and Methods

### 2.1. Study Design, Setting, and Subjects

As part of outreach efforts to enhance vaccination rates and the distribution of naloxone to under-resourced populations in central Indiana, over the past year, our pharmacy team has collaborated with local organizations to host community-based health access events. More recently, tobacco use screening and cessation interventions were layered on top of these existing healthcare services. Specifically, this evaluation was conducted at eleven health access events across two food pantries and one homeless shelter in Indianapolis, IN, between 15 June and 16 September 2022. To assess the demand for and interest in a tobacco cessation intervention, a cross-sectional survey was administered verbally by pharmacy team members as part of a broader intervention, which also involved the administration of vaccines (COVID-19 and influenza) at these sites.

### 2.2. Study Measures and Tobacco Cessation Interventions

A brief screening survey assessed individuals’ current use of tobacco products by asking, “Do you smoke cigarettes, use e-cigarettes, vape products, or any other type of tobacco?”. Those who reported current use were advised to quit and were asked, “Are you willing to quit in the next two months?”. Individuals who were interested in quitting within the next two months received brief cessation counseling that was provided by pharmacy personnel (i.e., pharmacists and/or pharmacy students) or other trained volunteers. Specifically, individuals were asked about their tobacco use and were advised to quit. An Indiana tobacco quitline resource card was offered to current tobacco users who were interested in quitting within the next two months. Acceptance of the card was recorded by the team member. This resource card included the number to call (1-800-QUIT-NOW) to enroll in a formal quitting program, which, in Indiana, includes multiple telephone-based sessions during which behavioral counseling is provided, along with information on medications. When available based on state resources, nicotine replacement therapy is provided at no cost and is mailed to individuals who are ready to quit and are at least 18 years of age. Medication options include the nicotine patch, nicotine gum, or nicotine lozenge. Callers who self-identify as having a mental health diagnosis are eligible to receive the nicotine patch plus the nicotine gum or nicotine lozenge (no physician’s authorization needed).

### 2.3. Data Collection and Analyses

At each event, anonymous data were collected by reading screening questions, and responses were logged prospectively into laptop computers by team members. Data were analyzed using standard descriptive statistics. Chi-squared tests of independence were used to identify group differences between clients of the food pantries and the homeless shelter, with an a priori *p*-value set at 0.05 for statistical significance. Data were analyzed using SPSS 28.0 [24]. These program evaluation data were deemed not to be a part of human subject research by the Purdue University Institutional Review Board.

## 3. Results

Over the three-month assessment period, eleven indoor mobile health access events were held across the three locations: two food pantries (seven events) and one homeless shelter (four events). Across 10 events at which vaccines were administered, a total of 122 COVID-19 and 144 influenza vaccines were administered (n = 266 total vaccines).

Combining data from all 11 events, 639 individuals were assessed for tobacco use (mean per event, 58; range, 7 to 141); 552 at a food pantry location, and 87 at the homeless shelter. An average of 79 and 22 individuals were assessed for tobacco use at each food pantry and homeless shelter event, respectively.

**Figure 1:**
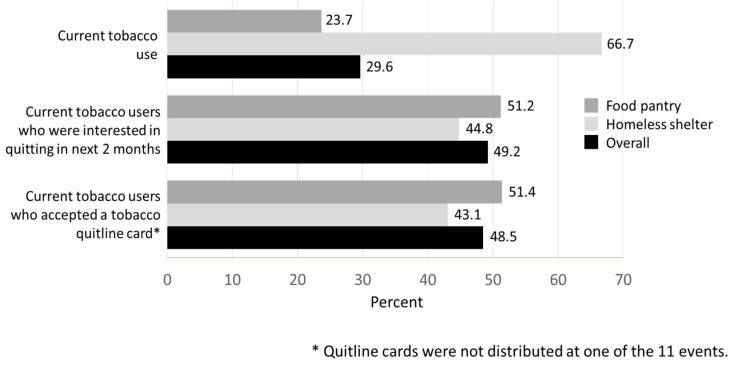
Current tobacco use, interest in quitting in the next two months, and tobacco quitline card acceptance (food pantry, homeless shelter, and overall; %).

When asked about tobacco use, 189 of the 639 individuals who were screened for tobacco use reported that they currently use tobacco (29.6%). The prevalence of tobacco use was significantly higher at the homeless shelter versus the food pantries (*p* < 0.0001; Figure 1). Of these 189 individuals who currently used tobacco, 93 (49.2%) indicated that they were interested in quitting in the next 2 months, and interest did not differ by location type (*p* = 0.42; Figure 1). Of the 85 individuals who expressed interest in quitting in the next 2 months, 79 (92.9%) accepted a tobacco quitline card. Acceptance of a quitline card also did not differ by location type (*p* = 0.31). The proportion of current tobacco users who accepted a quitline card, by location type and overall, is depicted in Figure 1.

## 4. Discussion

Because tobacco use disproportionately affects under-resourced communities [2,3,4,5], innovative models of care that are specifically designed to reach these populations are greatly needed. This initiative, led by pharmacists and pharmacy students, found that there was interest among clients receiving services at food pantries and homeless shelters for tobacco cessation assistance, indicating that it is feasible for pharmacy personnel to deliver brief cessation interventions in these settings.

Across eleven mobile health access events, approximately half of the clients currently using tobacco expressed interest in quitting within the next two months, and more than nine out of ten of these individuals accepted a quitline card. A lower percentage of individuals at food pantries were currently using tobacco; however, among those who smoked, interest in quitting did not differ significantly by site type (i.e., food pantries versus the homeless shelter). Despite the lower smoking prevalence, food pantries had much higher overall foot traffic and therefore afforded a greater potential to intervene with a larger number of individuals who use tobacco.

Prior research has similarly demonstrated the feasibility of introducing pharmacist-delivered tobacco cessation services in such settings. For example, Hartman-Filson and colleagues delivered smoking cessation interventions in homeless shelters by identifying and referring individuals who were interested in quitting to trained community pharmacists who assessed tobacco usage, provided counseling, prescribed nicotine replacement therapy, and arranged for the delivery of medications to the sites. In total, 84% of clients used a smoking cessation medication over the course of the study, and the average daily cigarette consumption decreased by 50%. Furthermore, weekly quit attempts increased from 28% to 47%. The authors concluded that partnerships between homeless shelters and community pharmacists improve access to counseling and medications for the treatment of tobacco use and dependence [19].

The role of pharmacists in providing preventive health services has evolved in recent years, and evidence suggests that services delivered by pharmacy personnel can serve to enhance individuals’ access to essential care [25]. For example, it has been established that pharmacists are key vaccination providers in the community; in one study, it was estimated that the percentage of older adults receiving influenza vaccines at a community pharmacy more than doubled between 2008 and 2015 [26]. Further, pharmacies have served as primary vaccination sites in the United States throughout the COVID-19 pandemic, with more than 301.1 million COVID-19 vaccines being administered through the Federal Retail Pharmacy program as of March 2023 [27]. As such, a parallel intervention that layers brief tobacco cessation counseling on top of existing vaccination and or other health services should be implemented and evaluated within community pharmacies.

As one of the most accessible healthcare settings, pharmacies can serve as a familiar space for individuals to discuss tobacco cessation with a trained healthcare provider, and the setting may provide additional opportunities to reach some populations that are under-resourced [28,29]. Simply asking about tobacco use, advising people to quit, and providing a brief intervention can be effective in encouraging individuals to start thinking about quitting [30]. Individuals who are ready to quit can either be referred to the tobacco quitline, a group program, or a web-based program, or they could be provided with support from a pharmacist as part of a tobacco cessation service. This type of brief intervention (asking about tobacco use, advising patients to quit, and referring those who are ready to quit to the available resources) can be achieved in less than a minute and has been shown to be effective in a variety of healthcare settings, including, but not limited to, pharmacies [20]. Connecting individuals directly with the tobacco quitline (as opposed to simply providing the quitline’s information and advising the individual to call the quitline) has been shown to further increase the odds of enrolling in the quitline service 13-fold, and this more proactive approach should be considered in future studies [21].

Through legislative advances, pharmacists in 16 states are now permitted to prescribe tobacco cessation medications for their patients [31]. As such, individuals in these states have enhanced access to evidenced-based methods as they begin their quit attempt. Pharmacists who prescribe cessation medications are also required to either provide behavioral counseling and/or refer their patients to other resources. These resources include the tobacco quitline and other evidence-based cessation programs.

Limitations of this demonstration project include the narrow geographic scope and the potential for bias due to the self-reported nature of the data. Despite these limitations, the findings of the current study suggest that mobile health access events can provide opportunities for pharmacists, pharmacy students, and other vaccine administrators to interface with individuals who use tobacco from populations that are under-resourced, assess their interest in quitting, and provide brief cessation interventions. Importantly, the project leveraged pharmacists and pharmacy students to deliver tobacco cessation interventions in conjunction with vaccination services, and thus no increase in manpower was needed beyond that which was already scheduled.

Continued efforts to scale up tobacco cessation interventions within food pantries and homeless shelters should be designed to overcome challenges that arise while serving under-resourced groups, as recommended by the Centers for Disease Control’s Cessation in Tobacco Prevention and Control Guidelines for Priority Populations [32]. For example, populations at both homeless shelters and food pantries might have poor literacy and health literacy skills [33]. To address this challenge, supplementary verbal information is needed to complement written educational content. Language barriers that exist between the pharmacy team and the food pantry clientele are addressable by hiring interpreters and assuring that quitline resources, and any other materials used, are translated into the appropriate languages. Lastly, quitline services are not a viable option for clientele with limited or no phone access [19]. Therefore, implementing a “one-stop shop” approach, which includes assistance with calling the quitline, the provision of brief cessation counseling, and the distribution of NRT on-site or via mail delivery, will help mitigate the barriers related to inconsistent follow-up.

In summary, these data support the feasibility of food pantries and homeless shelters as sites for identifying and connecting tobacco users with resources for quitting tobacco. The data also support the concept of layering tobacco use on top of other existing interventions. Future studies should include an assessment of client engagement with tobacco quitline and quit rates among those who accepted a quitline card during a community-based event. Lastly, while our project specifically focused on pharmacy personnel, it is important to note that the layering of cessation discussions on top of other healthcare services is clinician-agnostic, meaning that any trained professional (e.g., physician, physician associate/physician assistant, nurse, dentist, dental hygienist, social worker, etc.) could work in partnership with community organizations to provide similar events.

## Data Availability

The research data are not publicly available.
